# Tuberculosis (TB) of the Porta Hepatis Presenting with Obstructive Jaundice Mimicking a Malignant Biliary Tumor: A Case Report and Review of the Literature

**DOI:** 10.1155/2018/5318197

**Published:** 2018-12-05

**Authors:** Rashid AL Umairi, Ahmed AL Abri, Atheel Kamona

**Affiliations:** Department of Radiology, The Royal Hospital, Muscat, Oman

## Abstract

Localized hepatobiliary tuberculosis (TB) is a rare disorder which can present with an obstructive jaundice mimicking other noninfectious causes such as cholangiocarcinoma. Here, we report a case of porta hepatis tuberculosis in 19-year-old female who presented with an obstructive jaundice, and her computed tomography (CT) of the abdomen revealed a hepatic hilar mass with radiological features mimicking a malignant biliary tumor. We also review the literature related to this disorder.

## 1. Introduction

Mycobacterium tuberculosis is a contiguous and potentially fatal disease that usually affects the lungs. However, it can be extra-pulmonary in 15-20% of the cases of which abdominal tuberculosis (TB) accounts for 11-15% and hepatic TB for less than 1% [1–8]. Extra-pulmonary tuberculosis can be associated with pulmonary tuberculosis or it can rarely present as a localized disease, like in our case.

The clinical and radiological manifestations of hepatobiliary TB can be mistaken for malignant lesions leading to erroneous clinical diagnosis and unnecessary surgical intervention. We report a case of localized TB of the porta hepatis in a 19-year-old female who presented with a clinical picture of an obstructive jaundice. CT abdomen and pelvis showed a hepatic hilar mass with features suggestive of a malignant lesion that was further evaluated with a liver magnetic resonance imaging (MRI). In addition to the redemonstration of the hepatic hilar mass, MRI revealed features of liver microabscesses which were not visible in the CT scan. The additional findings from the MRI pointed toward TB of the porta hepatis as an alternative differential diagnosis. Our case report highlights the importance of considering TB of the porta hepatis within the differential diagnosis of liver hilar mass in a young patient with obstructive jaundice, and to our knowledge it is the first case to demonstrate the usefulness of MRI as a problem-solving tool to suggest preinterventional diagnosis.

## 2. Case Report

A 19-year-old Omani female not known to have any significant medical history was referred to our hospital with a history of upper abdominal discomfort more localized to the epigastric region and associated with jaundice and dark urine. There was no history of fever or night sweat nor history of travel. On physical examination, the patient was jaundice; otherwise, the systemic examination was unremarkable.

Complete blood count was within normal limits with a normal white blood count (6.3 10*∗*g/L). Liver function test revealed a picture of obstructive jaundice with a total bilirubin of 52 umil/L, Alkaline phosphatase 302 [iU] /L, and Alanine transaminase 457 [iU]/L. QuantiFERON-Tb gold test was positive.

**Figure 1:**
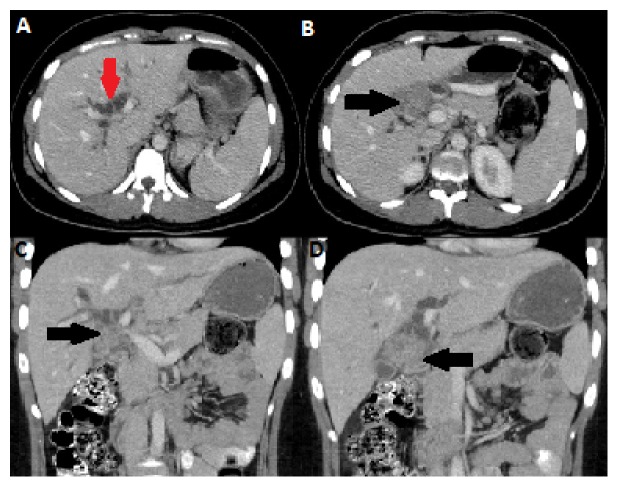
Enhanced CT scan of the abdomen axial (A, B) and coronal (C, D) images demonstrate an enhancing mass in the region of the porta hepatis (black arrow) with biliary duct dilatation (red arrow).

**Figure 2:**
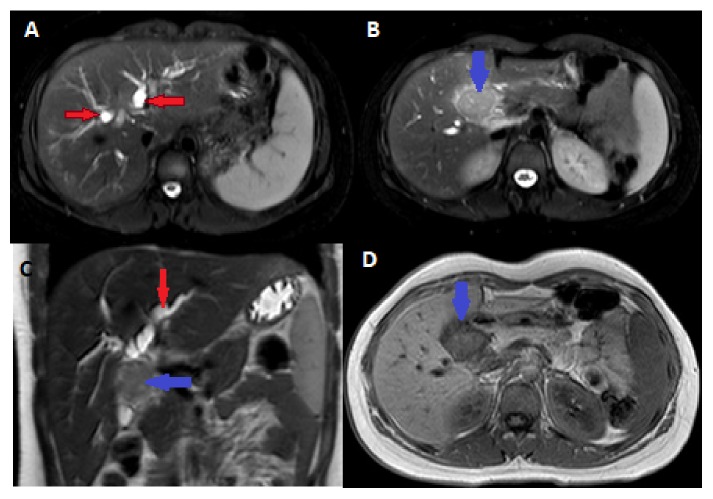
Axial (A, B) and coronal (C) T2-weighted images show a hyperintense mass in the region of the porta hepatis (blue arrow) with a dilated biliary ducts (red arrow). The mass (blue arrow) is hypointense in T1-weighted images (D).

**Figure 3:**
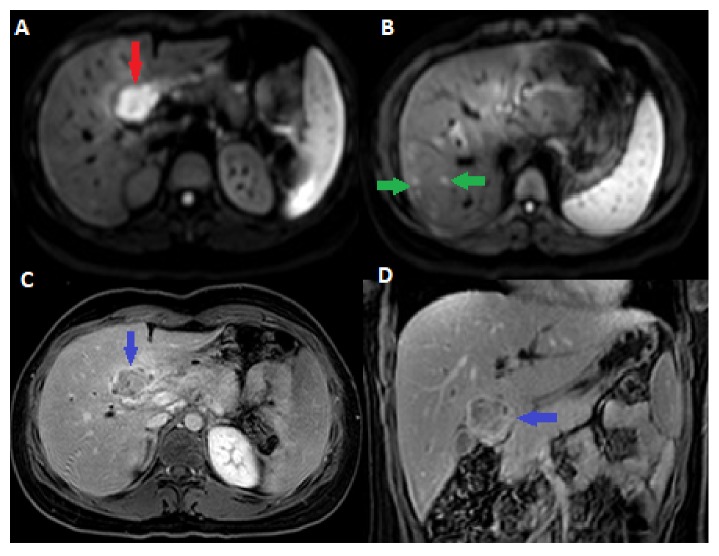
MRI of the liver axial diffusion-weighted (A, B) demonstrates a hyperintense mass in the region of the porta hepatis (red arrow) with a several hyperintense foci (green arrows). Axial postcontrast images (C, D) show heterogonous enhancement of porta hepatis mass (blue arrows).

CT scan of the abdomen and pelvis showed a lobulated and heterogeneous liver hilar mass with a central necrosis, measuring 2.4 x 3.9 cm. The mass was obstructing the proximal common hepatic duct resulting in dilatation of the intrahepatic biliary tree (Figure 1). The mass was associated with multiple enlarged peripancreatic, porta hepatis and hepatoduodenal lymph nodes, measuring up to 1.2 cm. None of the lymph nodes were showing central necrosis. Features were suggestive of a cholangiocarcinoma of the common hepatic duct. Further work-up with a liver MRI redemonstrated the porta hepatis mass. The mass was T2 hyperintense and T1 hypointense and showed moderate enhancement on postcontrast sequence with severe diffuse restriction (Figures 2 and 3). On MRCP, the mass was causing severe narrowing of the proximal 1.8 cm of the common hepatic duct, reaching the confluence and causing moderate dilation of the intrahepatic biliary tree. In addition, the MRI revealed multiple foci of restriction scattered throughout the liver and some of them showed subtle enhancement on postcontrast sequence suggestive of liver microabscess (Figure 3).Constellation of MRI findings was suggestive of localized TB of the porta hepatis; however, neoplastic lesion such as rhabdomyosarcoma and lymphoma could not be excluded and further evaluation with a tissue biopsy was advised. Chest radiograph was normal. Tumor markers were within normal limits including Cancer Ag 19-9 (CA 19-9): 8U/mL (range, 0-37U/mL), Carcinoembryonic antigen (CEA): 1.2 ug/L (range, 0-3ug/L), Alpha fetoprotein: 1.1 ug/L (range 0-15), and Chromogranin A: 47ug/L (range 26-92ug/L)

The patient underwent laparoscopic biopsy and was found to have enlarged porta hepatis lymph nodes. Biopsy was taken from the porta hepatis mass and histopathological examination showed a granulomatous inflammatory process. Although, no definite proof was obtained by culture or polymerase chain reaction (PCR), probable TB diagnosis was made based on the histological and imaging findings, and the patient was treated for 6 months with Rifampicin 150 mg and Isoniazid 75 mg. The patient showed significant response to treatment with dramatic improvement of liver function test. A follow-up liver function test 3 months after starting anti-TB medication showed normalization of Alkaline phosphatase 91 [iU] /L along with improvement of total bilirubin of 24 umil/L and Alanine transaminase 211 [iU]/L. After completion of anti-TB medication, liver function test was back to normal with a total bilirubin of 17 umil/L, Alkaline phosphatase 58 [iU] /L, and Alanine transaminase 16 [iU]/L. A follow-up CT abdomen was performed 10 months later and showed complete resolution of porta hepatis mass and intrahepatic biliary dilatation along with significant regression of previously noted enlarged upper abdomen lymph nodes.

## 3. Discussion

Obstructive jaundice caused by tuberculosis is a rare disorder which can mimic other noninfectious causes and can be overlooked due to low incidence. Unlike diagnosis of pulmonary TB, establishing the diagnosis of extra-pulmonary tuberculosis can be challenging due to nonspecific clinical symptoms and radiological findings.

Patient with abdominal TB can present with abdominal pain, low-grade fever, hepatosplenomegaly, and loss of weight and appetite [4, 9]. Biliary tuberculosis can present with jaundice. Four different mechanisms have been described in the literature as causes of obstructive jaundice secondary to hepatobiliary tuberculosis: porta hepatis TB lymphadenitis causing extrinsic compression of the common bile duct [5, 8, 10–13], head of pancreas involvement mimicking a pseudoneoplasm and obstructive the distal common bile duct [10, 14–18], a retroperitoneal mass caused by TB obstructing the distal bile duct [19], and a direct involvement of biliary epithelium or pericholangitis resulting in a single or multiple strictures mimicking cholangiocarcinoma [20–22]. Intrahepatic biliary involvement is usually due to hepatic granuloma and usually results in multiple intrahepatic biliary strictures [23]. Our patient had porta hepatis lymphadenitis compressing and obstructing the common hepatic duct.

Biological clues to the presence of biliary TB are not specific. Patients with biliary TB can have an impaired liver function test with a cholestasis picture secondary to obstruction, mimicking other noninfectious causes such obstructive biliary stone, primary sclerosing cholangitis, and a malignant cholangiocarcinoma [13].

Radiological diagnosis of biliary tuberculosis can be a challenging diagnosis with variable imaging findings ranging from bile duct thickening, biliary dilatation, and strictures to complete biliary obstruction [24]. On ultrasound, hypoechoic or rarely hyperechoic nodular lesion may be visualized in the porta hepatis region which is usually associated with biliary dilatation. On computed tomography (CT), the appearance varies from a hypodense mass with or without a postcontrast rim enhancement to a heterogeneous density with a necrotic center which can be associated with biliary dilatation [7, 8, 23, 24]. Baik et al. reported a hepatic hilar mass with central calcification which was encasing the portal vein and associated with biliary tree dilatation [8]. Miliary calcification along the bile duct which can be seen in ultrasound (US) and CT has been described as characteristic finding in biliary cholangitis [24]. In our case, CT showed a hepatic hilar heterogeneous mass encasing and narrowing the proximal common bile duct resulting in intrahepatic biliary dilatation.

Concomitant radiological features of TB elsewhere such as pulmonary or nodal involvement can help to narrow the differential diagnosis. In one series, chest radiographic changes consistent with pulmonary TB were observed in 4 out of 14 patients with hepatobiliary TB [9]. In our case, chest radiograph was normal with no findings suggestive of pulmonary tuberculosis.

Magnetic resonance cholangiopancreaticography (MRCP) is noninvasive imaging modality which has better tissue characterization than the US and the CT. In hepatic tuberculosis, lesions appear hypointense on T1-weighted images and hypointense, isointense, or hyperintense lesions with a peripheral hypointense rim on T2-weighted images depending upon the stage of the disease [24]. In our case, MRI of the liver revealed liver lesions with features suggestive of liver microabscess that are likely representing tuberculomas.

The definite diagnosis of hepatobiliary tuberculosis requires histopathologic evidence of caseating granuloma or demonstration of acid fast bacilli (AFB) on smear or culture of biopsy specimen. This can be achieve by obtaining a tissue sample by laparoscopy or endoscopic ultrasound with fine needle aspiration (FNA) [12]. Positive AFB smear in hepatic tuberculosis is low, ranging from 0% to 45%, and only 10% of cultures show a positive result. Therefore, AFB negative should not divert from the diagnosis, especially in areas where TB is endemic [4, 25]. Our patient underwent diagnostic laparoscopy and tissue biopsy revealed granulomatous disease, which responded to antituberculous therapy which supported the diagnosis of biliary TB.

## 4. Conclusion

Localized hepatobiliary TB is a rare disorder which can mimic other noninfectious causes. The current case highlights the importance of considering TB of the porta hepatis within the differential diagnosis of liver hilar mass in a young patient with obstructive jaundice, and the usefulness of MRI for suggesting preinterventional diagnosis.
